# Development of Asthmatic Response upon Bronchial Allergen Challenge Is Associated with Dynamic Changes of Interleukin-10-Producing and Interleukin-10-Responding CD4+ T Cells

**DOI:** 10.1007/s10753-014-9927-9

**Published:** 2014-05-28

**Authors:** Marcin Moniuszko, Kamil Grubczak, Krzysztof Kowal, Andrzej Eljaszewicz, Malgorzata Rusak, Marta Jeznach, Ewa Jablonska, Milena Dabrowska, Anna Bodzenta-Lukaszyk

**Affiliations:** 1 Department of Allergology and Internal Medicine, Medical University of Bialystok, Sklodowskiej-Curie 24A, 15-276 Bialystok, Poland; 2 Department of Regenerative Medicine and Immune Regulation, Medical University of Bialystok, Waszyngtona 13, 15-269 Bialystok, Poland; 3 Department of Immunology, Medical University of Bialystok, 15-274 Bialystok, Poland; 4 Department of Hematological Diagnostics, Medical University of Bialystok, 15-274 Bialystok, Poland

**Keywords:** asthma, allergen challenge, regulatory T cells, interleukin-10, interlukin-10 receptor, interleukin-7 receptor

## Abstract

The role of adaptive immune system in regulation of asthmatic responses remains elusive. Here, we performed a comprehensive time-course analysis of mutual relationships between development of asthmatic response following allergen challenge and changes in several CD4+ T cell subsets which we characterized as either releasing interleukin-10 (CD4+CD25−CD127− and CD4+CD25+CD127+ T cells) or responding to IL-10 (CD4+ T cell subsets expressing CD210). Patients that developed asthmatic reaction were described as responders (R) whereas the others were named non-responders (NR). In R, in contrast to NR, at 6 h, we demonstrated significant expansion of CD4+CD25−CD127− T cells which was followed by drop to baseline values at 24 h. In contrast, in R, we observed decrease in numbers of CD4+CD25+CD127+ and CD4+CD25−CD127+ T cells at 24 h. Interestingly, at baseline, despite comparable IL-10 levels, R presented with lower levels of all CD4+ T cell subsets expressing CD210. In R, the numbers of CD4+CD210+ T cell subsets were further decreased following bronchial challenge which was paralleled by decrease in IL-10 serum levels. Altogether, our data suggest that dynamic interactions between IL-10-producing and IL-10-responding CD4+ T cells could contribute to pathogenesis of asthmatic responses in atopic individuals.

## INTRODUCTION

CD4+ T cells play crucial roles in regulation of immune responses underlying allergic inflammation [1]. In our previous study, we highlighted importance of natural regulatory CD4+ T cells (Treg) in modulation of asthmatic response in allergic individuals subject to intrabronchial allergen challenge [2]. We demonstrated that lack of bronchoconstriction following specific allergen bronchial provocation is associated with transient decrease in the number of circulating putative natural regulatory CD4+ T cell phenotype, namely, CD4+CD25+CD127−. Interestingly, we did not demonstrate any changes in the number of CD4+CD25+CD127− T cells in patients who developed classical asthmatic reaction in response to bronchial allergen challenge. This might suggest that other immune mechanisms that are not related to natural Treg cells could account for increased susceptibility to the development of bronchoconstriction in atopic individuals. Indeed, correlations between allergic inflammation underlying asthmatic responses and alterations of adaptive immune system have not been investigated in sufficient detail. Development of asthmatic response upon bronchial allergen challenge was related previously to decrease in the number of circulating IL-4- and interferon (IFN)-gamma-producing CD4+ T cells [3]. On the other hand, enhancement of IL-10 production by peripheral CD4+ T cells in patients developing asthmatic response upon bronchial allergen challenge was demonstrated by Matsumoto *et al.* [4]. To date, it remains unknown whether differential clinical responses to allergen challenge could be also related to dynamic quantitative changes of other CD4+ T cell subsets, including adaptive Treg cells or non-regulatory CD4+ T cells. In fact, reports comprehensively describing simultaneous alterations of different T cell subsets and data on mutual correlations among them in response to allergen challenge are scarce. One of the major obstacles for precise *ex vivo* delineation and/or isolation of adaptive Treg cells is the need for *in vitro* stimulation of T cells and subsequent intracellular analysis of secreted cytokines, e.g., IL-10. Recently, however, the use of combination of anti-CD25 and anti-CD127 antibodies has allowed for precise delineation of not only natural Treg cells (characterized by CD4+CD25+CD127− phenotype with high expression of FoxP3), but also putative adaptive Treg cells, namely CD4+CD25−CD127− T cells. This subset was demonstrated to possess suppressive features despite the absence of CD25 expression and low expression of FoxP3 [5–7]. Further studies confirmed that CD4+T cells lacking both CD25 and CD127 did not produce Th1-, Th2-, and Th17-associated cytokines but secreted high amounts of IL-10 [8]. On the other hand, dynamic changes in distribution of CD4+ T cells with non-regulatory phenotype (mostly positive for CD127) were not analyzed in the context of development of asthmatic responses in sensitive subjects exposed to specific allergen.

Given the crucial role of IL-10-mediated actions in regulation of allergic inflammation, we performed here a comprehensive simultaneous analysis of those circulating CD4+ T cells that are capable of either secreting IL-10 or responding to IL-10 [9, 10]. We first set out to investigate which CD4+ T cell subsets previously described as putative adaptive Treg cells negative for CD127 (CD4+CD25−CD127− T cells) or non-regulatory CD4+ T cells positive for CD127 (CD4+CD25+CD127+ and CD4+CD25−CD127+ T cells) bear the highest potential for production of immunosuppressive IL-10 [11]. For comparison, we assessed which CD4+ T cell subsets are most capable of releasing Th1-characteristic cytokine exerting opposite to IL-10 effects, namely, IFN-gamma. We aimed to analyze whether bronchial exposure to allergen is associated with alterations of these CD4+ T cells with varying capacities to secrete IL-10 and IFN-gamma. We correlated these data with time-course changes in IL-10 serum levels and absolute numbers of CD4+ T cells subsets capable of responding to IL-10-mediated signals, namely, CD4+ T cells bearing CD210 (IL-10R). Finally, we wished to investigate whether changes in intensity of allergen-induced airway inflammation assessed by exhaled nitric oxide measurement could be related to alterations of varying regulatory and non-regulatory CD4+ T cells. Altogether, we presented here a novel pattern of dynamic interactions between CD4+ T cell subsets capable of producing IL-10 and those that could respond to its actions mediated by IL-10R.

## METHODS

### Patients

We recruited 27 individuals with a history of dyspnea and cough following exposure to house dust and with skin prick test reactivity to *Dermatophagoides pteronyssinus* (Dp) and *Dermatophagoides farinae* (Df). The patients recruited for the study were non-smokers, had no history of other medical conditions, and had not been receiving any treatment for asthma before the study.

### Intrabronchial Allergen Challenge

All subjects were challenged intrabronchially with Dp extract according to a protocol described elsewhere [2]. Patients who developed asthmatic response (which was defined as at least 20 % fall of forced expiratory volume in 1 s) after bronchial challenge with Dp were defined as responders (R) in contrast to non-responders (NR). There were 18 R and 9 NR. EDTA anti-coagulated blood was drawn before and 6 and 24 h after allergen challenge. The study protocol was approved by local ethics committee.

### Exhaled Nitric Oxide Measurements

Assessment of nitric oxide (NO) concentration in the exhaled air was performed using chemiluminescence analyzer NOA™ 280i (Sievers, USA), according to the American Thoracic Society recommendations as described previously [12]. Exhalation time and flow were 30 s and 50 ml/s, respectively, for the air exhaled by patients from the total lung capacity. The pressure and exhalation flow was maintained in proper range by continuous monitoring of these parameters during the test.

### Cell Culture

EDTA anti-coagulated whole blood samples obtained from additionally recruited eight healthy volunteers were used to isolate peripheral blood mononuclear cells (PBMCs) by density gradient sedimentation. PBMCs were cultured in presence of 2 μl of phorbol 12-myristate 13-acetate (PMA)/ionomycine/brefeldin A (Leukocyte Activation Cocktail, BD Bioscience). PBMC cultured in medium alone (RPMI-1640 supplemented with 10 % fetal bovine serum and 1 % penicillin/streptomycin) served as a negative control. Following incubation for 6 and 24 h at 37 °C in a humidified 5 % CO_2_ atmosphere, the cells were harvested and washed immediately in phosphate-buffered saline (Biomed Lublin) to prepare the cells for extracellular and intracellular staining described below. Cell death ratio following 24-h culture assessed with the use of trypan blue was 2.5 % for unstimulated samples and 9 % for samples stimulated with Leukocyte Activation Cocktail.

### Immunostaining and Flow Cytometric Analysis

**Fig. 1:**
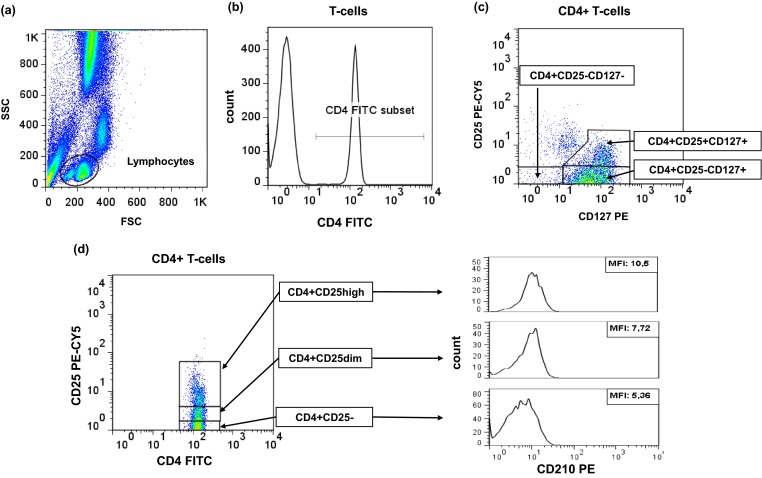
Flow cytometric plots characteristic for studied individuals. Representative FACS plots demonstrating gating strategy for identification of lymphocytes (**a**), CD4+ T cells (**b**), and their subpopulations, namely, CD4+CD25−CD127+, CD4+CD25+CD127+, and CD4+CD25−CD127− (**c**) and CD4+CD25−, CD4+CD25dim, CD4+CD25high T cells, and their CD210 expression (**d**). Precise delineation of all studied CD4+ T cell subsets was based on fluorescence-minus-one controls (e.g., staining only with anti-CD4 and anti-CD25, but not anti-CD127 monoclonal antibodies, or staining only with anti-CD4 and CD127, but not anti-CD25 monoclonal antibodies).

EDTA anti-coagulated whole blood samples or PBMCs collected after cell culture were stained with 5 μl of the following monoclonal antibodies: anti-CD4 FITC (BD Biosciences), anti-CD127 PE (Beckman Coulter), CD210 PE (BD Biosciences), anti-CD25 PE-Cy5 (BD Biosciences), and incubated for 30 min at room temperature, in the dark. For additional intracellular staining, the cells were permeabilized and incubated with monoclonal antibodies directed at either IL-10 (BD Biosciences) or IFN-gamma (BD Biosciences). Flow cytometric data were acquired on FACSCalibur flow cytometer (BD Biosciences, San Jose, CA, USA) and analyzed with the use of CellQuest software and FlowJo software as described previously [13]. *Ex vivo* data are presented as absolute numbers of CD4+CD25−CD127−, CD4+CD25−127+, and CD4+CD25+CD127+ (Fig. 1a–c) and CD4+CD25negCD210+, CD4+CD25intCD210+, and CD4+CD25highCD210+ T cells (Fig. 1d) per microliter of whole blood. Data acquired from *in vitro* culture are presented as a proportion of CD4+CD25−CD127− or CD4+CD25−CD127+ or CD4+CD25+CD127+ T cells releasing IL-10 or IFN-gamma after stimulation with PMA/ionomycine.

### Enzyme-Linked Immunosorbent Assay

Evaluation of IL-10 concentrations in sera of patients subject to bronchial allergen challenge was conducted using commercially available ELISA kit (Diaclone, France). BIO-RAD microplate reader (Model 550) was used to read the ELISA plates at 450-nm wavelength.

### Statistical Analysis

Following assessment of the data, non-parametric Wilcoxon test was used to evaluate statistical significance of the obtained results. The Spearman’s rank correlation coefficient was used to examine correlations. Statistically significant results were identified by a *p* value of <0.05 for all assays performed.

## RESULTS

### Phenotypical Characterization and Identification of IL-10-Producing CD4+ T Cell Subsets with Varying Levels of CD25 and CD127 Expression

**Fig. 2:**
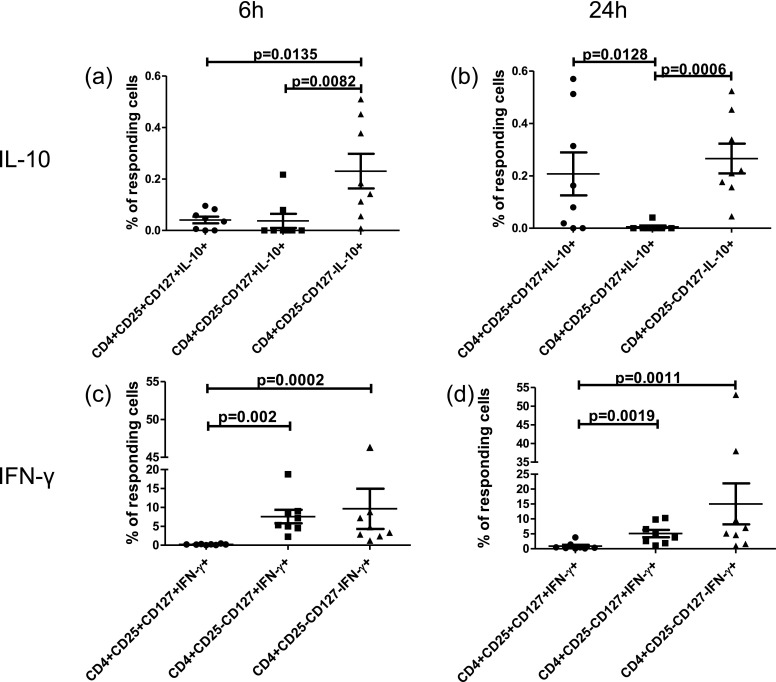
Assessment of capacity to produce IL-10 and IFN-γ by different CD4+ T cell subsets with varying levels of CD25 and CD127 expression. The summary of analyses of IL-10 (**a**, **b**) and IFN-γ (**c**, **d**) production by diverse CD4+ T cell subsets upon 6 (**a**, **c**) and 24 h (**b**, **d**) of PMA/ionomycine stimulation. The results are presented as a fraction (percentage) of cytokine-producing cells within entire CD4+ T cell population. In the *in vitro* experiments, the mean number of events acquired in CD4+CD25−CD127−, CD4+CD25−CD127+, and CD4+CD25+CD127+ gates was 9,989, 8,506, and 1,794, respectively.

Assessment of CD25 and CD127 expression serves as a sensitive marker for delineation of natural regulatory T cells characterized by CD4+CD25+CD127− phenotype. In contrast, much less is known about the other CD4+ T cell subsets characterized by varying levels of CD25 and CD127 expression. In this paper, we started with analyzing the functional potential (with regard to IL-10 and IFN-gamma secretion) of other than nTreg cell subsets, namely, CD4+CD25+CD127+, CD4+CD25−CD127+, and CD4+CD25−CD127− T cells (Fig. 2). We demonstrated that, following 24-h PMA/ionomycine stimulation, IL-10 is secreted by CD4+CD25−CD127− and CD4+CD25+CD127+, but not CD4+CD25−CD127+ T cells (Fig. 2b). Interestingly, at 6 h of stimulation, only CD4+CD25−CD127− T cells were the only subset producing IL-10 (Fig. 2a). These results are in part in concert with previous reports showing that CD4+ T cells lacking CD25 and CD127 are major producers of IL-10 [8]. In some contrast, CD4+CD25−CD127− T cells, but not CD4+CD25+CD127+, were producing highest levels of IFN-gamma (both at 6 and 24 h, Fig. 2c–d). Similarly, CD4+CD25−CD127+ T cells (that were negative for IL-10) were found positive for IFN-gamma. Thus, CD4+CD25−CD127−T cells turned out to produce both IL-10 and IFN-gamma, whereas CD4+CD25+CD127+ and CD4+CD25−CD127+ T cells appeared to produce either IL-10 or IFN-gamma, respectively.

### Dynamic Changes of CD4+ T Cell Subsets with Varying Levels of CD25 and CD127 Expression Following Bronchial Allergen Challenge

**Fig. 3:**
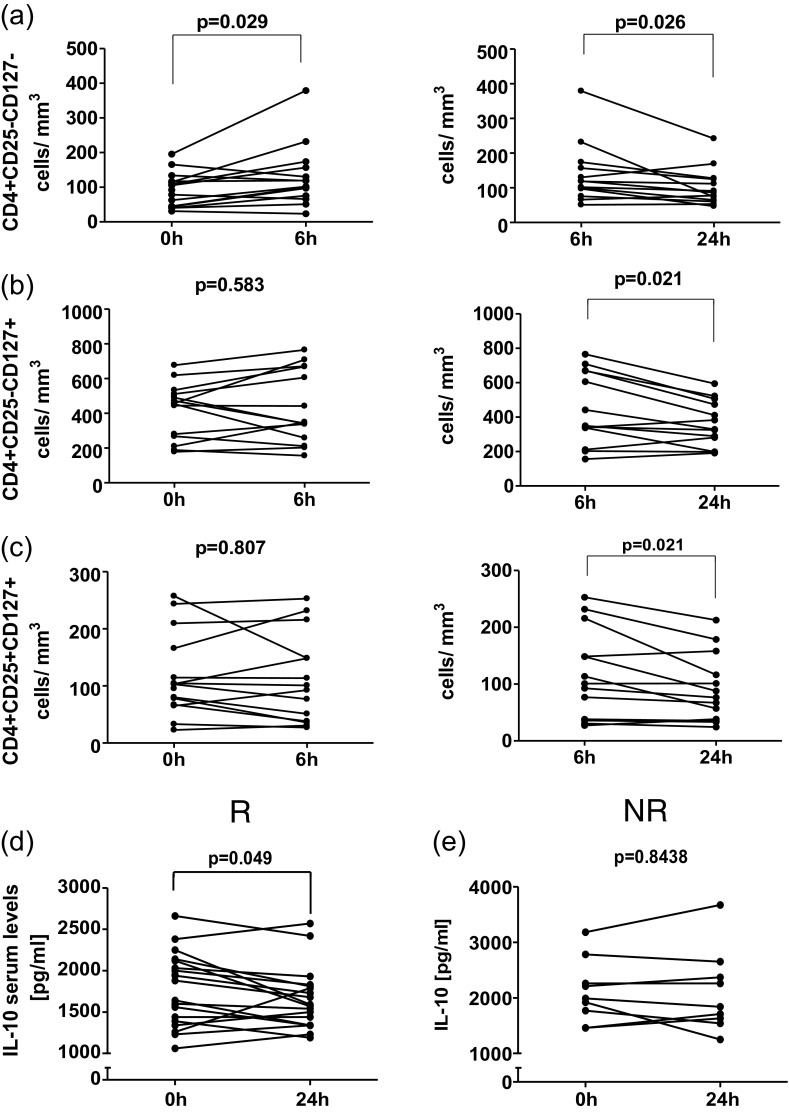
Time-course changes in absolute numbers of CD4+ T cell subsets characterized by varying levels of CD25 and CD127 expression (in R). (**a**, **c**) The summary of analyses of changes in absolute numbers of CD4+CD25−CD127− (**a**), CD4+CD25−CD127+ (**b**), and CD4+CD25+CD127+ (**c**) T cells at 0 versus 6 h (*left column*) and 6 versus 24 h (*right column*) after allergen challenge. **d**–**e** The summary of analyses of changes in IL-10 serum concentrations at 0 versus 24 h after allergen challenge in R (**d**) and NR (**e**).

Having found differential functional capacities of CD4+CD25−CD127−, CD4+CD25+CD127+, and CD4+CD25−CD127+ T cells, we next wished to investigate whether development of asthmatic response following bronchial allergen challenge could be associated with changes in absolute numbers of circulating diverse CD4+ T cell subsets. First, we observed that baseline numbers of these CD4+ T cell subsets were similar in R and NR (*p* = 0.279 for CD4+CD25−CD127− T cells, *p* = 0.526 for CD4+CD25+CD127+ T cells, and *p* = 0.192 for CD4+CD25−CD127+ T cells). Further, we demonstrated that each of the above-mentioned CD4+ T cell subsets presented with differential pattern of redistribution after bronchial allergen challenge. We demonstrated substantial increase in the number of CD4+CD25−CD127− T cells in R, but not NR (Fig. 3a). In R, at 6-h frequencies of CD4+CD25−CD127− increased significantly from 92.38 cells/mm^3^ [25th; 75th percentile, 44.49; 116.7] to 110.2 cells/mm^3^ [73.08; 161.2] (*p* = 0.029). At 24 h, the number of CD4+CD25−CD127− T cells in R dropped significantly and returned to values similar to those observed at baseline (from 118.6 cells/mm^3^ [86.38; 165.5] to 86.48 cells/mm^3^ [62.77; 125.9], *p* = 0.026). In contrary, no significant changes in the number of CD4+CD25−CD127− T cells were observed in NR (data not shown). Opposite pattern of changes was demonstrated for CD4+CD25+CD127+ and CD4+CD25−CD127+ T cells of which numbers dropped significantly after 24 h (but not 6 h) following development of asthmatic response (from 100.8 cells/mm^3^ [37.00; 182.3] to 76.40 cells/mm^3^ [36.77; 137.3], *p* = 0.021 and from 349.5 cells/mm^3^ [274.3; 669.9] to 328.5 cells/mm^3^ [240.2; 490.0], *p* = 0.021, respectively) (Fig. 3b, c). Again, no significant changes were found for CD4+CD25+CD127+ and CD4+CD25−CD127+ T cells in NR (data not shown).

### Dynamic Changes in IL-10 Serum Levels Following Development of Asthmatic Response During Bronchial Allergen Challenge

Having found changes in numbers of CD4+ T cells capable of secreting IL-10, we next wished to investigate changes in circulating IL-10 levels in the course of bronchial allergen challenge. At baseline, we did not find significant differences in IL-10 serum levels between R and NR (1,760 pg/ml [1,403; 2,098] and 1,990 pg/ml [1,615; 2,520], *p* = 0.150, respectively). However, at 24 h following bronchial allergen challenge, we found significant decrease in IL-10 serum levels in these individuals who developed asthmatic response (R) (from 1,760 pg/ml [1,378; 2,125] to 1,580 pg/ml [1,340; 1,815], *p* = 0.049) (Fig. 3d). Again, no difference in IL-10 levels was found in NR (Fig. 3e).

### Decreased Levels of CD4+ T Cells Expressing IL-10R in R

**Fig. 4:**
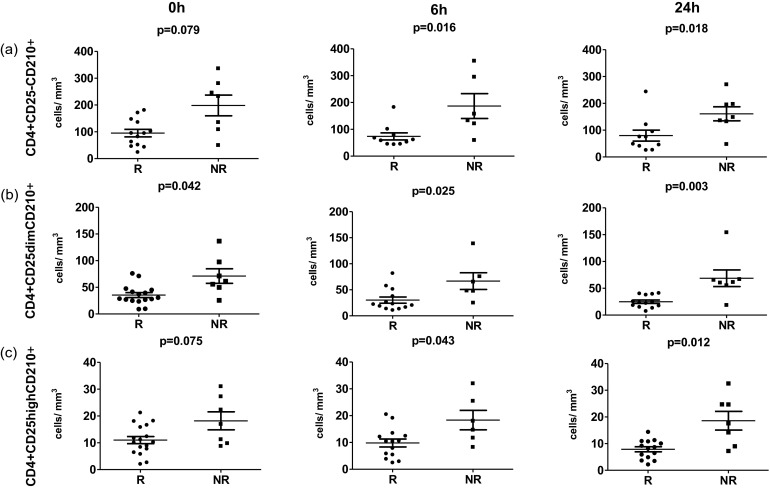
Quantification of changes of CD4+ T cell subsets expressing CD210 in the course of intrabronchial allergen challenge. The summary of analyses of numbers of CD4+ T cells expressing varying levels of CD25 and CD210, namely, CD4+CD25−CD210+ (**a**), CD4+CD25dimCD210+ (**b**), and CD4+CD25highCD210+ (**c**).

Next we set out to analyze whether changes in circulating IL-10 levels could be associated with alterations of CD4+ T cells capable of responding to actions mediated by this cytokine, namely, cells expressing IL-10R (CD210). Interestingly, at baseline, at 6 and 24 h, absolute numbers of CD4+CD210+ T cells were significantly lower in R than in NR (time 0, 106.4 cells/mm^3^ [65.65–152.2] vs 309.6 cells/mm^3^ [154.4–333.4], *p* = 0.005; 6 h, 68.29 cells/mm^3^ [57.45; 119.6] vs 166.6 cells/mm^3^ [135.6; 381.6], *p* = 0.021; 24 h, 81.18 cells/mm^3^ [53.04; 111.8] vs 197.2 cells/mm^3^ [178.3; 285.6], *p* = 0.009). This pattern was observed for all CD4+ T cell subsets expressing varying levels of CD25, namely, CD4+CD25−, CD4+CD25dim, and CD4+CD25high T cells (Fig. 4a–c). Furthermore, in R, but not NR, numbers of all CD4+ T cells positive for CD210 decreased over time and were significantly lower at 24 h as compared to those of baseline values (from 102 cells/mm^3^ [68.19; 146.3] to 81.18 cells/mm^3^ [53.04; 111.8], *p* = 0.018).

### IL-10 Levels Are Positively Correlated To Numbers of IL-10R-Positive CD4+ T Cells but Negatively to the Intensity of IL-10R Expression

We further investigated whether serum IL-10 levels are correlated to levels of CD4+ T cells expressing receptor for IL-10 (CD210). Notably, absolute numbers of CD4+CD25dimCD210+ and CD4+CD25highCD210+ T cells in R were positively correlated to serum IL-10 levels at each time point, with the exception of 24 h (Table 1). We then set out to analyze whether similar pattern can be observed for mean fluorescence intensity of CD210. However, in contrast to analysis of absolute numbers of CD210+ cells, mean fluorescence intensity of this receptor on CD4+CD25dim and CD4+CD25high was negatively correlated to serum IL-10 levels at 24 h (Table 2). In NR, baseline levels of serum IL-10 were positively correlated to absolute numbers of CD4+CD25highCD210+ T cells (Table 1), while no correlation between IL-10 levels and mean fluorescence intensity of CD210 in studied T cell subpopulations was found (Table 2).

**Table 1:** Correlations Among IL-10 Serum Levels and Absolute Numbers of CD4+CD210+ Subpopulations

		R		NR	
Time (h)	Population	*p* value	*r*	*p* value	*r*
0	CD4+CD25−CD210+	0.115	0.707	0.782	−0.126
	CD4+CD25dimCD210+	**0.025**	**0.539**	0.556	0.288
	CD4+CD25highCD210+	**0.001**	**0.696**	**0.006**	**0.937**
6	CD4+CD25−CD210+	0.310	0.357	0.950	0.100
	CD4+CD25dimCD210+	**0.002**	**0.758**	0.683	0.300
	CD4+CD25highCD210+	**0.003**	**0.727**	0.083	0.900
24	CD4+CD25−CD210+	0.472	0.276	1.000	−0.028
	CD4+CD25dimCD210+	0.249	0.360	1.000	−0.028
	CD4+CD25highCD210+	**0.001**	**0.789**	0.279	0.542

Bold values denote statistically significant correlations

**Table 2:** Correlations Among IL-10 Serum Levels and Mean Fluorescence Intensity of CD210 on Diverse CD4+ T Cell Subsets with Varying Levels of CD25 Expression

		R		NR	
Time (h)	Parameter	*p* value	*r*	*p* value	*r*
0	CD210 MFI of CD4+CD25−	0.916	−0.030	0.461	−0.311
	CD210 MFI of CD4+CD25dim	0.644	−0.116	0.500	−0.275
	CD210 MFI of CD4+CD25high	0.227	−0.299	0.461	−0.311
6	CD210 MFI of CD4+CD25−	0.711	−0.131	0.802	−0.142
	CD210 MFI of CD4+CD25dim	0.982	−0.006	0.919	−0.085
	CD210 MFI of CD4+CD25high	0.764	−0.088	0.802	−0.142
24	CD210 MFI of CD4+CD25−	0.712	−0.133	0.115	−0.619
	CD210 MFI of CD4+CD25dim	**0.022**	−**0.532**	0.096	−0.642
	CD210 MFI of CD4+CD25high	**0.019**	−**0.613**	0.115	−0.610

Bold values denote statistically significant correlations

### Positive Correlations Among Putative IL-10-Secreting and IL-10-Responding CD4+ T Cell Subsets

**Fig. 5:**
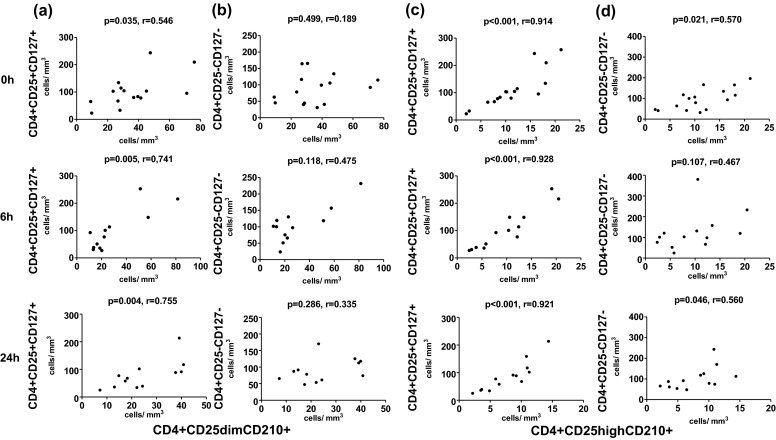
Correlations between putative IL-10-producing CD4+ T cells and CD4+ T cell subsets expressing CD210 (in R). The summary of correlation analyses of absolute numbers of CD4+CD25dimCD210+ and CD4+CD25+CD127+ (**a**) or CD4+CD25−CD127− (**b**) T cells or absolute numbers of CD4+CD25highCD210+ and CD4+CD25+CD127+ (**c**) or CD4+CD25−CD127− (**d**) T cells at 0 h (*upper line*), 6 h (*middle line*), and 24 h (*lower line*).

We next searched the pattern of mutual relationships between putative IL-10-producing CD4+ T cells and those CD4+ T cell subsets that express surface receptor for IL-10. We demonstrated that, in R, numbers of CD4+CD25dimCD210+ were positively correlated with CD4+CD25+CD127+ (Fig. 5a), but not CD4+CD25−CD127− T cells (Fig. 5b) at each time point. Moreover, absolute numbers of CD4+CD25highCD210+ were positively correlated with CD4+CD25+CD127+ (Fig. 5c) and CD4+CD25−CD127− (Fig. 5d) T cells (at each time point). On the other hand, we did dot observe any significant correlation in NR.

### Lack of Correlations Among Exhaled Nitric Oxide Levels and Levels of IL-10-Producing and IL-10-Responding CD4+ T Cell Subsets

We finally wished to investigate whether numbers of circulating both IL-10-producing and IL-10-responding CD4+ T cell subsets can be correlated to intensity of allergic airway inflammation routinely measured by assessment of nitric oxide concentrations in exhaled air. However, none of the studied CD4+ T cell subsets was found directly related to the levels of exhaled nitric oxide in atopic subjects (Table 3).

**Table 3:** Correlations Between Exhaled Nitric Oxide Concentrations and Analyzed CD4+ T Cell Subsets

	R		NR	
T cell subpopulation	*p* value	*r*	*p* value	*r*
CD4+CD25−CD127−	0.594	0.250	1.000	0.500
CD4+CD25+CD127+	0.906	0.071	0.333	1.000
CD4+CD25−CD127+	0.138	0.642	1.000	−0.500
CD4+CD25−CD210+	0.782	−0.142	0.159	−0.968
CD4+CD25dimCD210+	0.839	−0,107	0.333	1.000
CD4+CD25highCD210+	0.661	0.214	0.333	1.000

## DISCUSSION

Here, we described a novel pattern of dynamic changes of circulating CD4+ T cells with both regulatory and non-regulatory phenotypes in the context of development of asthmatic response following bronchial allergen challenge. First, we extended our knowledge on functional characteristics of CD4+ T cells with varying levels of expression of two crucial for CD4+ T cell homeostasis cytokine receptors, namely, CD127 and CD25. To date, according to scarce reports, CD4+ T cells positive for CD127 were considered non-regulatory in contrast to regulatory CD127-negative CD4+ T cells: natural CD4+CD25+CD127− and adaptive CD4+CD25−CD127− T cells [5]. Here, we further characterized the functional capacities of CD127-negative and CD127-positive CD4+ T cell subsets and confirmed in part findings of Liu *et al.* and Couper *et al.* who were referring to CD4+CD25−CD127− T cells as putative Tr1 cells, as these cells were shown to secrete IL-10 [5, 8]. Interestingly, capability to release IL-10 upon stimulation was not conferred only to CD4+CD25−CD127− T cells, as we demonstrated that similar feature is characteristic also for CD4+CD25+CD127+ T cells (Fig. 2). Notably, the latter subset was capable of selective IL-10, but not IFN-gamma production. Nonetheless, according to many reports, any classifications of human CD4+ T cell subsets to any Th subclass need to be taken with a great caution [14–17]. This notion is also supported by our current data demonstrating that putative Tr1 CD4+CD25−CD127− T cells secreting IL-10 are also capable of high IFN-gamma production rather being a feature of Th1 cells [18]. Importantly, our results indicate that neither CD25 nor CD127 expression is exclusively related to capability to secrete IL-10. Therefore, in the current report, we decided to precisely describe and present phenotype of studied CD4+ T cells rather than refer to them as to either Tr1 or Th1 cells.

Here, we used well-standardized allergen bronchoprovocation model because despite its laborious character it provided us a unique opportunity for observing dynamic interactions between allergen-induced bronchoconstriction and alterations of immune system [19–21]. Previously, using the same model, we demonstrated that changes in numbers of CD4+ T cells with natural regulatory phenotype were associated with protection from development of bronchoconstriction following bronchial allergen challenge [2]. In contrast, here, we found that circulating CD4+CD25−CD127− (putative IL-10- and IFN-gamma-producing) T cells were significantly enriched during allergen challenge in individuals with asthmatic reaction (Fig. 3). It remains unknown whether this change represents mobilization of potential anti-inflammatory cells that could counterbalance the effects of asthmatic airway inflammation. Interestingly, the nature of these interactions seems more complex than expected as we demonstrated here that intensity of allergic airway inflammation assessed by measurements of exhaled nitric oxide is not directly correlated to the numbers of circulating CD4+ T cell subsets. Regardless the mechanism, comparison of our data on putative adaptive regulatory CD4+ T cells with those published previously on natural regulatory CD4+ T cells suggests that both subsets may play differential roles in orchestrating immune responses accounting for development of airway hyperreactivity.

In addition, our data indicate that enhanced mobilization of circulating putative IL-10-secreting and IFN-gamma-secreting CD4+ T cells (putative Tr1+Th1 T cells) is not sufficient to inhibit development of asthmatic reaction in allergic individuals. Based on previous reports, we could speculate that coexistence of Tr1 and Th1 lineages in single CD4+ T cell subset could result in functional diminishment of either Tr1- or Th1-like functions [20]. Moreover, we demonstrated that asthmatic reaction is accompanied by significant drop in serum levels of IL-10 (Fig. 3d). One could hypothesize that this drop in serum IL-10 levels was caused by lack of mobilization (and even significant drop) of CD4+CD25+CD127+ T cells, selectively secreting IL-10, but not IFN-gamma. It is possible that therapeutic strategies aimed at enhancement of these CD4+CD25+CD127+ T cells being selective IL-10 producers could be of potential benefit in immune modulation of asthmatic response. Altogether, our data suggest that airway allergen challenge is counteracted by mobilization of certain (but not all) IL-10-dependent anti-inflammatory mechanisms that, on the other hand, are not sufficient to inhibit development of asthmatic response.

Notably, we demonstrated here that decreased IL-10 concentrations following bronchial allergen challenge are accompanied by decrease in absolute numbers of CD4+25dim IL-10R+ and CD4+CD25high IL-10R+T cells (Fig. 4). Interestingly, decreased number of CD4+ T cells expressing IL-10R was paralleled (before and 24 h after bronchial challenge) by enhancement of mean fluorescence intensity of this receptor on CD4+ T cells. This strong positive correlation suggests an existence of interesting axis between IL-10-producing and IL-10-responding CD4+ T cells: Limited amounts of IL-10 seem to be preserved for lesser number of CD4+IL-10R+T cells that in turn express higher levels of this receptor. Altogether, these data support the observation that local exposure to allergen affects trafficking of different T cell subsets and therefore is associated with systemic alterations of T cell-mediated immunity. This pattern does not seem to be restricted only to adaptive immune system, as we recently demonstrated that airway allergen challenge led to significant alterations of different monocyte subsets [22].

Recent reports indicated that the use of IL-10-targeted experimental therapies could provide protection against asthma in mouse model of this disease [9, 23, 24]. Our data confirm that existing IL-10-dependent mechanisms in atopic patients prone to development of asthma are not sufficient: These patients have lower numbers of CD4+ T cells positive for IL-10R, and in addition, their IL-10 levels drop following bronchial allergen challenge. Identification of these alterations provides another evidence that the need for further development of therapeutic anti-asthmatic strategies aimed at IL-10/IL-10R axis is still warranted. On the other hand, our observation of decreased baseline IL-10R levels in R suggests that determination of IL-10R on CD4+ T cells could become a useful predictor of occurrence of asthmatic response in atopic subjects. Thus, assessment of IL-10R on CD4+ T cells could help in prospectively identifying these atopic subjects who are at higher risk of developing bronchoconstrictive reactions.

## CONCLUSIONS

In summary, we presented here novel pattern of mutual interactions between CD4+ T cell subsets capable of producing IL-10 and those that could respond to its actions mediated by IL-10R in the course of bronchial challenge with specific allergen. In addition, our data suggest that strategies targeting IL-10/IL-10R axis could be helpful in alleviating intensity of bronchial responses following exposure to allergens.
